# Strategies to Reduce HIV Transmission amongst Prisoners; Lessons Learned From Iran

**Published:** 2017-07-17

**Authors:** Kamran Bagheri Lankarani, Nooshin Zarei, Hassan Joulaei

**Affiliations:** ^1^ Health Policy Research Center, Institute of Health, Shiraz University of Medical Sciences, Shiraz, Iran; ^2^ Shiraz HIV/AIDS Research Center, Institute of Health, Shiraz University of Medical Sciences, Shiraz, Iran

## Dear Editor-in-Chief

Transmission of blood-borne viruses is a global health issue amongst prisoners^1^. According to the national prison administration’s report, 225 624 people have been kept in prisons in Iran at December 2014 that shows a slight decrease in comparison with the year 2013^2^. Due to remarkable over-representation of injecting drug users (IDUs) among Iranian prisoners, HIV prevalence is 8 times higher in prisons than general population estimation^1, 3^. To control the epidemic of such disease, Iran’s Judiciary with the collaboration of the Ministry of Health (MOH), considered harm reduction programs since 2004. The implemented programs comprised the following items: Voluntary Counseling and Testing (VCT) for every inmate even after release, Triangular Clinics (TCs), Methadone Maintenance Therapy (MMT), condom distribution and providing conjugal visit rooms in prisons. During the time MOH has strengthened its active surveillance system among prisoners to monitor the outcome of the interventions.

As a result, the number of HIV cases has decreased slowly but continually and reached 3,700 cases in 2012. Evidently, HIV prevalence among prisoners has decreased more quickly than any other high-risk groups (such as FSWs and MSMs)^4^. It was due to high coverage of MMT and development of TC cervices^5, 6^, which in turn led to a gradual reduction in drug usage in prisons^7^.

In conclusion, applying a multi-sectorial planning model has led to remarkable success to control HIV amongst Iranian prisoners. Nevertheless, the third wave of HIV epidemic is worrisome in both inside and outside the prisons. The illegality of sex working and all types of extra-marital sex in Iran has resulted in the incarceration of these high-risk groups that could facilitate transmission of HIV between prison and community. Hence, it seems that the way forward could be more difficult than the way behind. Therefore, strengthening harm reduction programs and establishing an active follow-up system for prisoners with high-risk behavior or HIV after releasing from the prison should be considered more seriously. Furthermore, preparing and conducting comprehensive preventive and promoting programs to control high-risk behaviors amongst different groups of the population is necessary.

## Conflict of interest

The authors declared no conflict of interest.

Kamran Bagheri Lankarani (MD)^1^, Nooshin Zarei (MA)^2^, Hassan Joulaei (MPH, PhD)^2^*

^1^ *Health Policy Research Center, Institute of Health, Shiraz University of Medical Sciences, Shiraz, Iran*

^2^ *Shiraz HIV/AIDS Research Center, Institute of Health, Shiraz University of Medical Sciences, Shiraz, Iran*

*****Correspondence to:***** *Hassan Joulaei (MPH, PhD)*

***E-mail:*** *joulaei_h@yahoo.com*
